# Air pollution and kidney cancer risk: a systematic review and meta-analysis

**DOI:** 10.1007/s40620-024-01984-x

**Published:** 2024-06-24

**Authors:** Lina Dahman, Victoria Gauthier, Aurore Camier, Jean Joel Bigna, François Glowacki, Philippe Amouyel, Luc Dauchet, Aghiles Hamroun

**Affiliations:** 1 Service de Santé Publique, Epidémiologie, Economie de la Santé et Prévention, CHU de Lille, Lille, France; 2 Service de Santé Publique, Epidémiologie, Economie de la Santé Et Prévention, CHU de Lille, Lille, France; 3 UMR1167 RID-AGE, Institut Pasteur de Lille, Inserm, Univ Lille, CHU Lille, Lille, France; 4 Médecins du Monde, 93120 Saint-Denis, France; 5 Département de Santé Publique, Epidémiologie, Institut Pasteur du Cameroun, Yaoundé, Cameroun; 6 Service de Néphrologie, CHU de Lille, Lille, France; 7 Faculté de Médecine, Université Catholique de Lille, Lille, France

**Keywords:** Kidney cancer, Air pollution, Particulate Matter, Nitrogen dioxide

## Abstract

**Background:**

Although several risk factors of kidney cancer have already been well-addressed, many remain underappreciated, such as chronic exposure to air pollution. This systematic review and meta-analysis aims to assess the association between air pollutant exposure and the risk of kidney cancer.

**Methods:**

With an exhaustive search equation including keywords related to air pollution and kidney cancer on EMBASE, PubMed, Web of science, Cochrane Library and CINAHL database, we identified all relevant articles published before March 23rd, 2023 (Prospero registration number: CRD42020187956). Using random-effects meta-analysis, we present pooled hazard ratios (with their respective 95% confidence interval) associated with a 10 µg/m^3^ increase in each pollutant level. Heterogeneity was quantified by the I_2_ statistic. Risks of methodological and publication bias were also both assessed using appropriate tools.

**Results:**

Of the 1919 records identified, our review included 19 articles (13 cohort, 5 registry-based and 1 case–control studies), of which 9 were suitable for the meta-analysis. We found a significantly increased risk of kidney cancer incidence for a 10 μg/m^3^ elevation of both particulate matter of less than 10 µm (PM_10_) (HR = 1.29 [1.10; 1.51], *I*^2^ = 0%, *p* = 0.002) and nitrogen dioxide (NO_2_) (HR = 1.10 [1.03; 1.18], *I*^2^ = 20%, *p* = 0.004). Secondary analyses also suggest an increased risk of kidney cancer-related morbidity-mortality associated with PM_10_ exposure.

**Conclusions:**

Overall, our findings suggest a potential association between exposure to increased levels of PM_10_ and NO_2_ and the risk of kidney cancer. These results should nonetheless be interpreted with caution due to the limited number of included studies and their significant risk of methodological bias.

**Graphical abstract:**

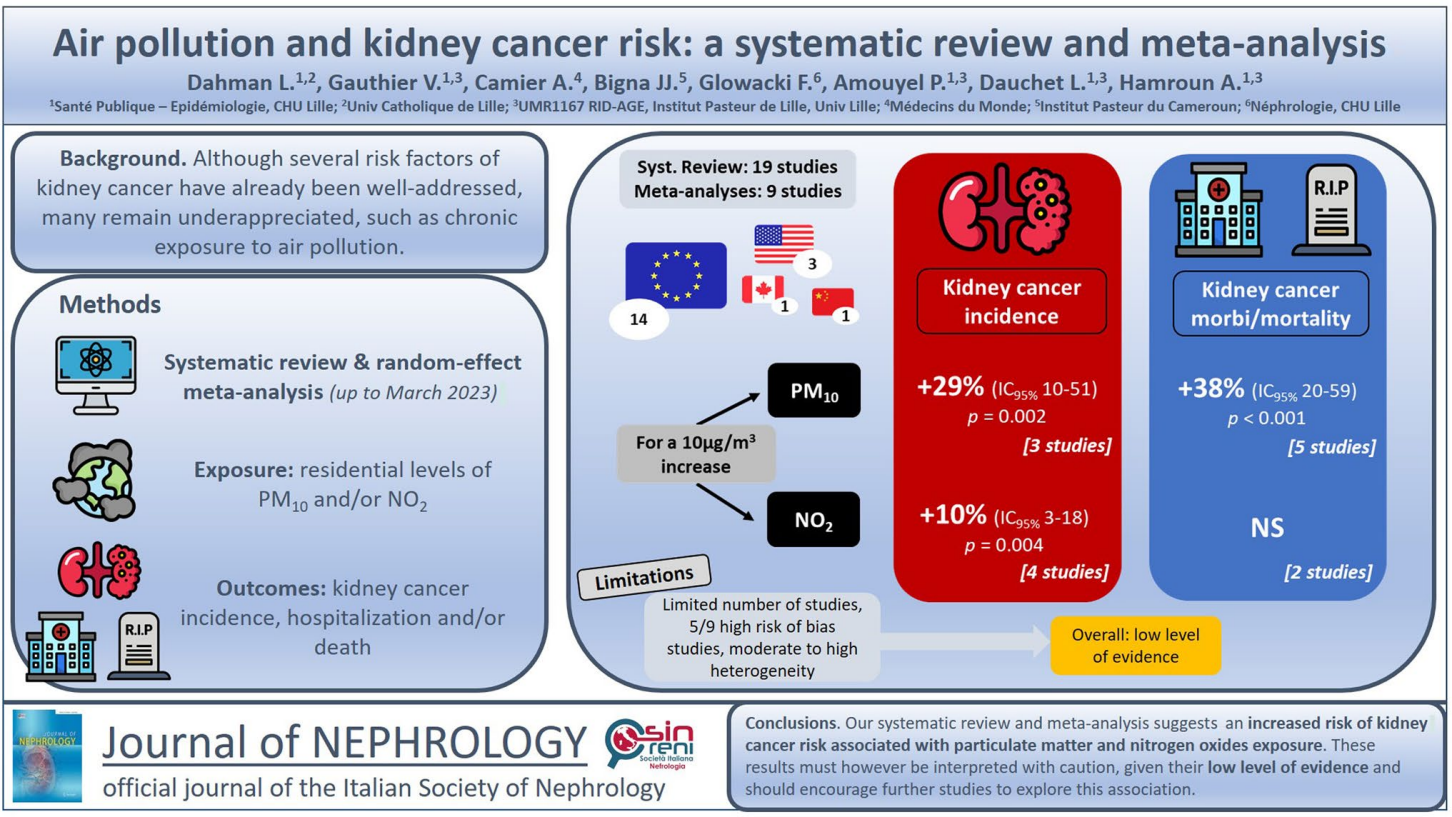

**Supplementary Information:**

The online version contains supplementary material available at 10.1007/s40620-024-01984-x.

## Introduction

Worldwide, in 2020, the number of new cases of kidney cancer was estimated at 431,000, and 80,000 people are estimated to die annually of kidney cancer. In the United States, kidney cancer is the sixth most common cancer for men and the ninth most common for women [1]. In addition to age, sex and genetic predisposition, several other risk factors have been associated with kidney cancer incidence and mortality. Smoking is associated with a two-fold higher risk of developing kidney cancer [2]. Overweight and obesity are responsible for 30% of kidney cancers in Europe and 13% of kidney cancer-related deaths [3]. However, some other potential risk factors are less studied, such as air pollution.

Air pollution is a complex mixture of gaseous compounds and suspended liquid or solid particles, mainly the result of human activity. Two commonly measured pollutants are known to be associated with deleterious effects on human health: nitrogen oxides (NO) (especially NO_2_) and particulate matter (PM_10_ and PM_2.5_). Previous meta-analyses have already highlighted an association between exposure to these pollutants and increased risk of all-cause mortality, as well as respiratory, cerebrovascular and cardiovascular mortality [4, 5]. Nitrogen dioxide is a toxic gas that forms in the atmosphere from nitric oxide, which is mainly produced by road traffic and industrial activities. As for particulate matter, they are mainly produced during combustion phenomena or formed by chemical reactions from precursor gases present in the atmosphere. Since the 1980s, a few epidemiological studies have evaluated the link between kidney cancer and exposure to exhaust gases from combustion vehicles within the general population [6–8] and within particularly exposed occupations, such as gas stations employees [9, 10], railway workers [11] or urban mail carriers [12]. However, these studies have reported conflicting results. Other population-based studies have shown an association between air pollution and kidney cancer risk, but through highly indirect and imprecise exposure assessments. Indeed, air pollution was approximated according to the proximity of the participants’ place of residence to a polluting source or according to their occupation. It was not until the 2010s that research started using data from monitoring stations providing approximate estimations of atmospheric compounds. A few studies have recently been published investigating the risk of kidney cancer in association with particulate matter [13–17] and nitrogen oxide [18–20] exposure but without reaching a clear overall conclusion on the issue.

The improvement of air pollution measurement methods, the already established proof of its deleterious effects on human health, and the need to deepen our knowledge of kidney cancer risk factors motivated the realization of this systematic review and meta-analysis. Our study hypothesis relies on the potential increased risks of kidney cancer related to higher levels of air pollutant concentrations.

## Methods

This systematic review was performed according to the Preferred Reporting Items for Systematic Reviews and Meta-Analyses (PRISMA) recommendations [21] and registered in Prospero (registration number: CRD42020187956). Two independent investigators (Li.D., A.H.) were responsible for the selection process of articles included in the systematic review and meta-analysis, involving identification, screening, and inclusion. Additionally, they were responsible for extracting and managing data, as well as assessing the risk of bias in the included studies. After completing each step independently, the two investigators (Li. D, A.H.) compared their results and resolved any discrepancy by discussion.

### Literature search strategy

This meta-analysis is part of a larger study investigating the association between air pollution and overall kidney health [22]. A systematic search was conducted within the following electronic databases: EMBASE, PubMed, Web of science, Cochrane Library and CINAHL database until March 23rd, 2023 (Supplementary Table 1). We screened the title and abstract of each retrieved study to exclude duplicates and irrelevant studies. Then, the references of the selected articles were consulted to identify other articles potentially meeting our inclusion criteria. Finally, the last screening step was based on full text articles. Additionally, the references of all relevant articles were scanned for other relevant data sources missed during our search.

### Eligibility criteria

All articles involving human beings which presented original data on the link between air pollution and kidney cancer and published in full text or meeting abstract were eligible for inclusion, with no restriction on study design, publication date, or language. We excluded animal studies, ex vivo and toxicological studies, commentaries and editorials, case reports, and studies with no original data. If a citation lacked enough quantitative data and these essential data could not be obtained from the corresponding author, the study was excluded. The diagnosis of cancer was made based either on medical records with clinically confirmed diagnosis or the International Classification of Diseases 10 code C64 (“Malignant neoplasm of kidney”) in medico-administrative databases. Air pollution exposure was defined as any method of air pollutant exposure measurement, including assessments of pollutant concentration by monitoring stations, use of satellite-based or land-use regression models, and use of indicators of long-term traffic exposure. Because air pollutant exposure was often presented as a continuous level regarding a specific population study, the generated effect was expressed for an appropriate standardized increase of air pollutant exposure (see “Data extraction and management” section).

### Quality assessment

The Newcastle–Ottawa Scale, with some modifications, was adapted to assess methodological quality in this specific context of environment-health association studies, according to validated scales in previous reports and the Cochrane Collaboration [23–25]. The overall risk of bias assessment is based on four sub-evaluations: exposure assessment bias, detection bias, selection bias and adjustment for confounders. The detailed process of the methodological quality assessment is provided in Supplementary Table 2.

### Data extraction and management

The bibliographic information for authors, publication year, sample size, follow-up time, outcome and details on exposure (nature of pollutant, method of assessment, level of increase) were extracted via a structured form developed for this study. For the quantitative analysis, we also extracted hazard ratio—and respective 95% confidence interval—of kidney cancer risk corresponding to the increased level of air pollutant. The PM_2.5_ concentrations were converted to PM_10_ and the NO_x_ concentrations to NO_2_ using the methods previously described [26, 27]. Then, all increases in concentration levels were brought to 10 μg/m^3^ based on the indications of the European Commission when conversions were necessary. Finally, the hazard ratios (and 95% confidence intervals) were standardized for a 10 µg/m^3^ increase in each pollutant concentration [25].

### Statistical analysis

We included all relevant studies in the qualitative analysis, regardless of the method used to estimate air pollution (emission sources or interpolation). In addition, studies that provided quantified estimates of exposure to air pollutants and measures of association were included in the meta-analysis for further quantitative analysis. We conducted random-effects meta-analysis using the DerSimonian and Laird method (integrating intra- and inter-study variation) [28] estimating the pooled effect size derived from (i) studies evaluating the association between each air pollutant exposure and the risk of kidney cancer incidence, and (ii) studies evaluating the association between each air pollutant exposure and the risk of morbidity-mortality due to kidney cancer (either kidney cancer-related hospitalization or death). The pooled results are expressed as hazard ratios with their 95% confidence intervals for the incidence, mortality and hospitalizations of kidney cancer associated with a 10 μg/m^3^ increase in the atmospheric concentration of PM_10_ or NO_2_. An overall summary of kidney cancer risk was also produced by combining the results of the three outcomes. When a study estimated the associations for several outcomes, only the estimation based on the highest number of events was included in the overall analysis. *P* < 0.05 was considered as statistical significance cut-off. Heterogeneity was assessed using the *Ꭓ*_2_ test from Cochran’s *Q* statistic and expressed with the I2 statistic [29]. Values of 25, 50, and 75% are used to define low, medium, and high heterogeneity, respectively. The presence of publication bias was assessed by Egger’s test [30] and visual inspection of the funnel plots [31]. Sensitivity analyses were also conducted by excluding studies at high risk of bias. Due to the expected low number of studies included in the various meta-analyses, subgroup analyses could not be performed. All statistical analyses were made using Comprehensive Meta-Analysis Version 4 software (Borenstein, M., Hedges, L., Higgins, J., & Rothstein, H. Biostat, Englewood, NJ 2022).

## Results

### Systematic review

A total of 1919 articles were retrieved using the research equation. After removing duplicates, we screened the titles and abstracts of the remaining studies, leading to the identification of 81 articles that investigated the potential association between air pollution and kidney cancer (Supplementary Fig. 1). Finally, we included 19 relevant studies in our systematic review, of which 9 were used for meta-analysis. We found 13 cohort studies [8–16, 18–20, 32], 5 registry-based studies [7, 17, 33–35] and 1 case control study [6] (Table 1). Most of the studies were conducted after 2001 (75%). Fourteen studies were carried out in Europe, four in North America and only one in Asia. Follow-up time ranged from 2 to 24 years and cohort sizes varied from 1393 to more than one million individuals.

**Table 1:** Summary of the included studies

Author, *Country (Year)*	Study design	Population	Exposure	Exposure assessment method	Outcome	Outcome assessment method	Adjustment factors	Main results
Siemiatycki J. et al., *Canada (1988)* [6]	Case–control	*N* = 3726 100% men	Exhaust and combustion products	Experts’ evaluation	Incidence of various types of cancers	Not specified	Age, ethnic group, socioeconomic status, smoking habits, “dirtiness” of the job, beverage consumption, marital status, other occupational exposures	No significant increase in incidence
Lagorio S. et al., *Italy (1994)* [10]	Prospective cohort, 11 years of follow up	*N* = 2665 Filling station managers Sex ratio ≈ 6:1	Gasoline vapors compounds	Workload indicators (employment duration, station size …)	All-cause mortality and cancer mortality	Registry linkage using the ICD ***	None	No significant increase in mortality
Lynge E. et al., *USA (1997)* [9]	Prospective cohort, 20 years of follow up	*N* = 19,000 Service station workers Sex ratio ≈ 7:1	Gasoline vapors compounds	Benzene levels (estimation)	Incidence of various types of cancers	Registry linkage using the ICD ***	None	Significant increase in incidence
Soll- Johanning H. et al., *Denmark* *(1998)* [11]	Retrospective cohort, 1900–1994	*N* = 18,174 Bus drivers and tramway employees Sex ratio ≈ 8:1	Vehicle exhaust fumes	Employment duration	Incidence of various types of cancers	Registry linkage using the ICD ***	None	Significant increase in incidence among men only
Michelozzi P. et al., *Italy (1998)* [33]	Ecological, 1987–1993	*N* = 2,775,000 Inhabitants of Rome	Industrial facilities emissions	Proximity to industrial sites	Cancer mortality	Registry linkage using the ICD ***	Socioeconomic level based on education, occupation, unemployment, number of family members, overcrowding, ownership of dwellings	Significant increase in mortality among women only
Boffetta P. et al., *Sweden (2001)* [7]	Ecological, 1971–1989	43 million person-years, workers	Diesel emissions	Estimation based on occupation	Incidence of various types of cancers	Registry linkage using the ICD ***	Age, rural or urban residence	Significant increase in incidence among men only
Soll- Johanning H. et al., *Denmark (2004)* [12]	Retrospective cohort, 1898–1996	*N* = 17,233 Urban mail carriers Sex ratio ≈ 5:1	Polycyclic aromatic hydrocarbon	1-hydroxy pyrene/ creatinine urinary ratio	Incidence of various types of cancers	Registry linkage using the ICD ***	None	No significant increase in incidence
Guo J. et al., *Finland (2004)* [8]	Retrospective cohort, 1971–1995	*N* = 1,180,231 Workers born between 1906 and 1945 Sex ratio ≈ 1:1	Gasoline (CO) or diesel (NO_2_) engine exhausts	Experts’ evaluation	Incidence of various types of cancers	National Cancer Registry	Socioeconomic status, cigarette smoking, body mass index, age, calendar period	No significant increase in incidence
García-Pérez J. et al., *Spain (2013)* [34]	Ecological, 1997–2006	8098 Spanish cities	Waste treatment plant emissions	Proximity to polluting sites	Cancer mortality	National records	Population size, percentage illiteracy, farmers and unemployed persons, average persons per household, and mean income	Significant increase in mortality
Cong X. et al., China (2018) [35]	Ecological, 1983–2010	*N* > 23 million Shanghai Cancer Registry Sex ratio ≈ 1:1	Industrial waste gas emissions, SO_2_ and soot	Statistics annual report from Shanghai Environmental Protection Bureau	Incidence of various types of cancers	Registry linkage using the ICD ***	Gender, number of doctors per 10,000 population (as a medical-social indicator), education and Engel coefficient (as a socioeconomic indicator)	Significant increase in incidence
*Studies included in the meta-analyses (quantitative exposure assessment)*								
Raaschou- Nielsen O. et al., *Denmark* *(2011)* [18]	Prospective cohort, 10 years of follow up	*N* = 53,404 from 50 to 64 years old Sex ratio ≈ 1:1	NO_x_	Danish AirGIS modeling system	Incidence of various types of cancers	Registry linkage using the ICD ***	BMI, smoking, hypertension, education, occupation	No significant increase in incidence
Ancona C. et al., Italy (2015) [13]	Retrospective cohort, 2001–2010	*N* = 85,559 Sex ratio ≈ 1:1	H_2_S, PM_10_, SO_X_	Lagrangian dispersion model + LUR*	Mortality and hospitalization rates of various types of cancers	Registry linkage using the ICD ***	Gender, age, education, occupation, civil status, area-based -socioeconomic position and outdoor NO_2_ concentration	No significant increase in hospitalizations nor mortality
Raaschou- Nielsen O et al., *Denmark* (2016) [14]	Prospective cohort, 14 years of follow up	*N* = 289,002	PM_2.5_, PM_10_, NO_2_, NO_x_	LUR*	Incidence of various types of cancers	Registry linkage using the ICD***	Age, sex, calendar time, smoking status/ intensity/ duration, occupation/employment status, educational level, BMI, hypertension, area-level socioeconomic status	No significant increase in incidence
Cohen G. et al., Israel (2016) [19]	Prospective cohort, 16 years of follow up	N = 1,393 Survivors of myocardial infarction < 65 years old Sex ratio ≈ 4:1	NO_x_	LUR*	Incidence of various types of cancers	Registry linkage using the ICD ***	Age, sex, ethnicity, socioeconomic status, obesity, smoking status	No significant increase in incidence
Turner M. et al., USA (2017) [15]	Prospective cohort, 22 years of follow up	*N* = 623,048 Age ≥ 30 years old Sex ratio ≈ 1:1	PM_2.5_, NO_2_ and O_3_	LUR* + Bayesian maximum entropy interpolation model	Cancer mortality (except lung cancer)	Death certificates using the ICD ***	Age, race/ethnicity, gender, education; marital status; body mass index smoking status, vegetable/fruit/fiber consumption, fat consumption; beer, wine, liquor consumption; industrial exposures; occupation dirtiness index; and ecological covariates	Significant increase in mortality
Gandini M. et al., *Italy (2018)* [16]	Prospective cohort, 7 years of follow up	*N* = 74,989 Age > 35 years old Sex ratio ≈ 1:1	PM_2.5_, NO_2_	Integrated Modeling system for atmospheric pollution	Various causes of hospitalization	Registry linkage using the ICD ***	Gender, educational level, marital status, occupational status, smoking habit, physical activity and BMI	Significant increase in hospitalizations
Cohen G et al., Israel (2018) [20]	Historical prospective cohort, 7 years of follow up	*N* = 9816 Post-PCI** Sex ratio ≈ 3:1	NO_x_	LUR*	Incidence of various types of cancers	Registry linkage using the ICD ***	Sex, smoking, neighborhood socioeconomic status, ethnicity, hypertension, diabetes mellitus, chronic heart failure, renal failure and hemoglobin levels	Significant increase in incidence
Coleman N. et al., USA (2020) [17]	Registry-based study	*N* > 8.5 million cancer patients Sex ratio ≈ 1:1	PM_2.5_	Integrated empirical geographic regression models	Incidence of various types of cancers	Registry linkage using the ICD ***	percentage male; percentage White, Black, Hispanic, and other; percentage who did not graduate high school, graduated high school, or obtained more education than high school; median income, rent, and home value; percentage below 150% poverty; percentage working class; percentage unemployed; percentage living in a rural area; percentage smokers; percentage who consume alcohol; percentage who are physically active; and percentage of individuals in a country who are obese	No significant increase in incidence
Hvidtfeldt U. and al, Denmark (2022) [32]	Pooled cohorts, 1985–2005	*N* = 302,493 from six European cohorts	PM_2.5_, NO_2_, black carbon, O_3_	LUR*	Incidence of various types of cancers	Registry linkage using the ICD ***	Age, sex, year of baseline visit, BMI, smoking status, duration, intensity, marital status, and employment status, 2001 mean income at the area level	No significant increase in incidence

* Land-use regression model

** Percutaneous coronary interventions

*** International Classification of Diseases

Different air pollutants or proxies of air pollution with different assessment approaches were used across the studies. Among the classic air pollutants, older studies reported mainly the exposure to vehicle exhaust fumes and/or combustion products (6 studies), assessed with participants’ occupations and work-load factors (e.g. length of employment, duration of shifts, and service station size or quantity of fuel sold for filling station attendants). Other articles used the exposure to industrial emissions (4 studies), which is assessed based on participants’ residency area (proximity to waste incinerator sites or power plants). In more recent studies, PM_10_, PM_2.5_, and NO_x_ were the most commonly used air pollutants (9 studies), with air pollution modeling based on land-use regression being the most frequent method used to estimate the exposure at the participant’s place of residence.

Most studies used data from national or regional cancer registries to detect outcome occurrence (15 studies), and some used hospital registries (2 studies) or death certificates (2 studies). Kidney cancer incidence, mortality and hospitalization were reported in respectively 13, 5 and 2 studies.

### Meta-analysis

All studies included in the meta-analysis were conducted in Europe or the United States, estimating exposure using land use regression models and identifying the outcome through the application of ICD codes on medical-administrative databases. Moreover, the majority of these studies accounted for age, sex, ethnicity, body mass index, smoking status, education level, and occupation type among the adjustment factors. Only one study considered other pollutants among the confounders.

**Fig. 1:**
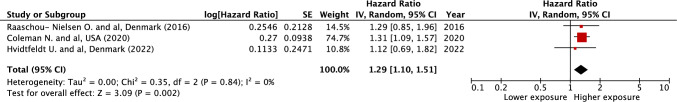
Association between PM_10_ exposure and the risk of kidney cancer incidence. *CI* confidence interval, *df* degrees of freedom, *SE* standard error

**Fig. 2:**
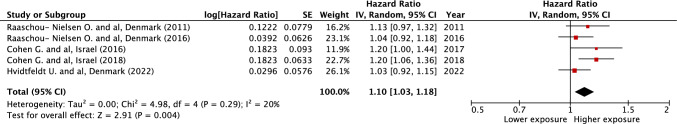
Association between NO_2_ exposure and the risk of kidney cancer incidence. *CI* confidence interval, *df* degrees of freedom, *SE* standard error

**Fig. 3:**
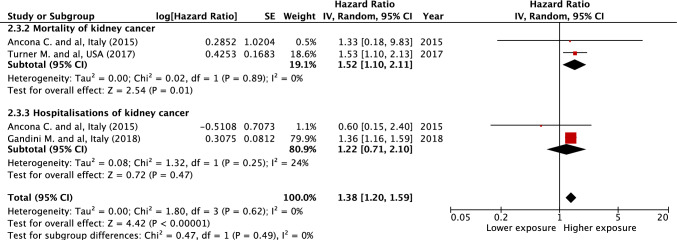
Association between PM_10_ exposure and the risk of kidney cancer-related morbidity/mortality (hospitalization or death). *CI* confidence interval, *df* degrees of freedom, *SE* standard error

**Fig. 4:**
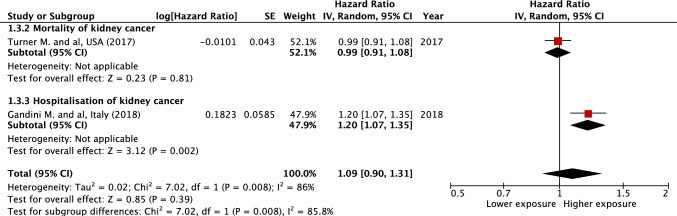
Association between NO_2_ exposure and the risk of kidney cancer-related morbidity/mortality (hospitalization or death). *CI* confidence interval, *df* degrees of freedom, *SE* standard error

The overall results suggest a significant association between PM_10_ or NO_2_ exposure and kidney cancer risk. We observed a significant increased risk of kidney cancer incidence for a 10 μg/m^3^ elevation in PM_10_ (HR = 1.29 [1.10; 1.51], *I*^2^ = 0%, *p* = 0.002) and NO_2_ (HR = 1.10 [1.03; 1.18], *I*^2^ = 20%, *p* = 0.004) (Figs. 1 and 2). Regarding the incidence of hospitalization and death for kidney cancer, the association is only observed for PM_10_ (HR = 1.38 [1.20; 1.59], *I*^2^ = 0%, *p* < 0.001 and HR = 1.09 [0.90; 1.31], *I*^2^ = 86%, *p* = 0.39 for a 10 μg/m^3^ elevation in PM_10_ and NO_2_, respectively) (Figs. 3 and 4). Overall, when considering all outcomes combined, the increased hazards of kidney cancer associated to air pollution exposure are globally similar to those of previous analyses, for a 10 μg/m^3^ elevation in PM_10_ (HR = 1.34 [1.20; 1.49], *I*^2^ = 0%, *p* < 0.001) and NO_2_ (HR = 1.10 [1.03; 1.17], *I*^2^ = 53%, *p* = 0.007) (Supplementary Figs. 2 and 3). The heterogeneity ranges from low for kidney cancer incidence analyses to very high for hospitalization and mortality analyses. Five of the nine studies included in the meta-analysis present an overall high risk of bias, mainly related to a partial adjustment for relevant confounders (Table 2). In sensitivity analysis excluding studies with a high risk of bias, the previously observed associations are no longer significant for kidney cancer incidence and persist in the analysis including all outcomes (Supplementary Figs. 4–9). No significant publication bias has been observed as suggested by visual inspection of the funnel plots (Supplementary Fig. 10), and Egger’s test one-tailed *p*-value > 0.10 for all analyses, irrespective of the air pollutant.

**Table 2:** Methodological bias assessment (studies included in the meta-analysis)

Author, Country (Year)	Exposure assessment bias	Detection bias	Selection bias	Adjustment for confounders	Global risk of bias
Raaschou-Nielsen O. et al., Denmark (2011)	High	High	Low	Low	High
Ancona C. et al., Italy (2015)	High	Low	Low	High	High
Raaschou-Nielsen O et al., Denmark (2016)	Low	Low	Low	Low	Low
Cohen G. et al., Israel (2016)	Low	Low	High	High	High
Turner M. et al., USA (2017)	Low	Low	High	Low	Medium
Gandini M. et al., Italy (2018)	Low	Low	Low	Low	Low
Cohen G et al., Israel (2018)	Low	Low	High	High	High
Coleman N. et al., USA (2020)	High	Low	Low	High	High
Hvidtfeldt U. et al., Denmark (2022)	Low	Low	Low	Low	Low

## Discussion

Overall, our study shows a significant increased risk of kidney cancer incidence associated with PM_10_ and NO_2_ exposure (+ 29% and + 10% for a 10 µg/m^3^-increase of each pollutant exposure, respectively). These results also suggest an increased risk of kidney cancer morbidity (hospitalization or death) associated with PM_10_ exposure. These findings should however be interpreted with caution due to the low level of the retrieved evidence.

To the best of our knowledge, this is the first attempt to provide a quantified synthesis for the association between air pollution and kidney cancer in the general population. In October 2020, Sakhvidi M. et al. published a systematic review on air pollution exposure and bladder, kidney and urinary tract cancer risk [36], but no meta-analysis was performed. The results of their review suggested a potential association between air pollution and an increased risk of urinary tract cancer. Moreover, the ESCAPE study, based on the data from 14 European cohorts, showed an increased risk of kidney cancer incidence related to PM_10_ exposure, albeit not statistically significant. Hvidtfeldt U. *et al* [32] pooled data of more than 300,000 individuals across Europe, and did not observe any increased risk of kidney cancer in association with long-term air pollution exposures of NO_2_, PM_2.5_, black carbon (BC), ozone (O_3_), or elemental components of PM_2.5_. However, the six European cohorts included in their study were selected to represent areas in the lower exposure range, whereas other studies reporting for higher exposure to PM_2.5_ tend to find mostly significant associations [36]. Furthermore, the data identified in our systematic review are mainly generated in Europe and North America, regions of the globe where exposure to air pollution remains moderate compared to Asian countries. These results potentially underestimate the excess risk that could be observed in regions more exposed to air pollution. Overall, the discrepant results in the literature are probably linked to two main factors that are particularly heterogeneous across studies: (i) the inclusion of tobacco among the confounding factors, which is highly inconsistent from one study to another, and (ii) the level of chronic exposure to air pollution, which varies greatly from one region of the globe to another.

The carcinogenic effect of air pollution on the kidney is currently understudied [37]. Since 2013, the International Agency for Research on Cancer (IARC) has recognized fine particulate matter from air pollution as a well-established carcinogen [38]. Nowadays, the carcinogenic impact of air pollution is clearly identified for lung cancer. Although less studied, other data suggest the carcinogenic potential for other solid cancers such as colon, brain, and breast cancer [39]. While the airways are the primary target for inhaled particles, evidence from animal studies show that ultrafine particles can translocate to other organs such as the liver, kidneys, heart and brain [40–43]. Diesel particles, in particular, have been shown to induce cancer-related processes in the kidneys, including oxidative stress [44] and DNA damage [45]. Additionally, exposure to particulate matter can lead to an angiotensin/bradykinin system imbalance [46], early kidney damage, and inflammation, ultimately contributing to the development of urological cancers. Other mechanisms have also been suggested, including cell membrane disruption, induction of pro-inflammatory cytokines along with tumor necrosis factor α, and pro-apoptotic signals [39]. The involvement of polycyclic aromatic hydrocarbons and benzo(α)pyrenes accompanying NO_2_ in vehicle emissions has also been suggested [47]. Chronic kidney disease, which has been observed in individuals exposed to particulate matter [48–51], is also associated with kidney and bladder cancer recurrence and progression [52, 53]. These findings suggest that exposure to air pollutants could contribute to a vicious circle favoring the onset of chronic kidney disease, itself a risk factor for kidney cancer. This effect could add to the potential carcinogenic effect of environmental exposure.

These results must be interpreted with caution, given their debatable level of evidence. This is due to the small number of studies included in the meta-analysis, the significant level of heterogeneity and the high risk of methodological bias in half of the studies. It should be noted, however, that the risk-of-bias evaluation grid is strict, leading to the identification of any methodological bias. Given the observational nature of the included studies, the probable residual confounding prevents any conclusion regarding a potential causal nature of the observed association. Furthermore, all included studies employed an indirect method for estimating exposure to air pollution, which may have led to misclassification bias. However, this bias is likely non-differential and results in an expected underestimation of the actual exposure. Unfortunately, the limited number of studies included in the meta-analysis does not allow us to explore the factors likely to explain the observed heterogeneity. However, although we cannot completely rule it out, the risk of publication bias seems moderate in this study. Although this would be of great interest, in this review we were unable to examine any effect modification due to a potential interaction with socioeconomic indicators. Future research perspectives not addressed in this review should focus on multi-environmental exposure as opposed to the silo approach most often employed in the literature, as well as individually assessing indoor air pollution, still little studied to date.

In conclusion, our systematic review and meta-analysis suggests an increased risk of kidney cancer risk associated with particulate matter and nitrogen oxide exposure. These findings should encourage further studies to explore this association and the specific kidney pro-carcinogenic mechanisms related to air pollution.

## Supplementary Information

Below is the link to the electronic supplementary material.

Supplementary file1 (PDF 120 KB)

**Supplementary Fig. 2**. Association between PM_10_ exposure and overall kidney cancer risk (all outcomes combined). *CI, confidence interval; df, degrees of freedom; SE, standard error* (PDF 530 KB)

**Supplementary Fig. 3**. Association between NO_2_ exposure and overall kidney cancer risk (all outcomes combined). *CI, confidence interval; df, degrees of freedom; SE, standard error* (PDF 515 KB)

**Supplementary Fig. 4**. Sensitivity analysis: association between PM_10_ exposure and the risk of kidney cancer incidence, excluding high risk of bias studies. *CI, confidence interval; df, degrees of freedom; SE, standard error* (PDF 179 KB)

**Supplementary Fig. 5**. Sensitivity analysis: association between NO_2_ exposure and the risk of kidney cancer incidence, excluding high risk of bias studies. *CI, confidence interval; df, degrees of freedom; SE, standard error* (PDF 180 KB)

**Supplementary Fig. 6**. Sensitivity analysis: association between PM_10_ exposure and the risk of kidney cancer-related morbidity/mortality (hospitalization or death), excluding high risk of bias studies. *CI, confidence interval; df, degrees of freedom; SE, standard error* (PDF 304 KB)

**Supplementary Fig. 7**. Sensitivity analysis: association between NO_2_ exposure and the risk of kidney cancer-related morbidity/mortality (hospitalization or death), excluding high risk of bias studies. *CI, confidence interval; df, degrees of freedom; SE, standard error* (PDF 310 KB)

**Supplementary Fig. 8**. Sensitivity analysis: association between PM_10_ exposure and overall kidney cancer risk (all outcomes combined), excluding high risk of bias studies. *CI, confidence interval; df, degrees of freedom; SE, standard error* (PDF 429 KB)

**Supplementary Fig. 9**. Sensitivity analysis: association between NO_2_ exposure and overall kidney cancer risk (all outcomes combined), excluding high risk of bias studies. *CI, confidence interval; df, degrees of freedom; SE, standard error* (PDF 433 KB)

**Supplementary Fig. 10**. Funnel plots (Trim and fill analysis) regarding PM_10_ (A) and NO_2_ (B) analyses. Unfilled and black filled circles correspond to the observed and imputed studies. Unfilled and black filled diamond shapes correspond to the observed and imputed point estimates (log hazard ratio). (PDF 136 KB)

Supplementary file11 (PDF 71 KB)

## Data Availability

All the data used for this study are already publicly available.

## References

[1] Kidney Cancer - Statistics. Cancer.Net. Published June 25, 2012. Accessed January 1, 2023. https://www.cancer.net/cancer-types/kidney-cancer/statistics

[2] Agudo A, Bonet C, Travier N et al (2012) Impact of cigarette smoking on cancer risk in the European prospective investigation into cancer and nutrition study. J Clin Oncol Off J Am Soc Clin Oncol 30(36):4550–4557. 10.1200/JCO.2011.41.0183 10.1200/JCO.2011.41.0183 23169508

[3] Pischon T, Lahmann PH, Boeing H et al (2006) Body size and risk of renal cell carcinoma in the European Prospective Investigation into Cancer and Nutrition (EPIC). Int J Cancer 118(3):728–738. 10.1002/ijc.21398 16094628 10.1002/ijc.21398

[4] Hamra GB, Guha N, Cohen A et al (2014) Outdoor particulate matter exposure and lung cancer: a systematic review and meta-analysis. Environ Health Perspect 122(9):906–911. 10.1289/ehp/1408092 24911630 10.1289/ehp/1408092 PMC4154221

[5] Orellano P, Reynoso J, Quaranta N, Bardach A, Ciapponi A (2020) Short-term exposure to particulate matter (PM10 and PM2.5), nitrogen dioxide (NO_2_), and ozone (O_3_) and all-cause and cause-specific mortality: systematic review and meta-analysis. Environ Int 142:105876. 10.1016/j.envint.2020.105876 32590284 10.1016/j.envint.2020.105876

[6] Siemiatycki J, Gérin M, Stewart P, Nadon L, Dewar R, Richardson L (1988) Associations between several sites of cancer and ten types of exhaust and combustion products. Results from a case-referent study in Montreal. Scand J Work Environ Health 14(2):79–90. 10.5271/sjweh.1949 2455336 10.5271/sjweh.1949

[7] Boffetta P, Dosemeci M, Gridley G, Bath H, Moradi T, Silverman D (2001) Occupational exposure to diesel engine emissions and risk of cancer in Swedish men and women. Cancer Causes Control CCC 12(4):365–374. 10.1023/a:1011262105972 11456233 10.1023/a:1011262105972

[8] Guo J, Kauppinen T, Kyyrönen P, Heikkilä P, Lindbohm ML, Pukkala E (2004) Risk of esophageal, ovarian, testicular, kidney and bladder cancers and leukemia among finnish workers exposed to diesel or gasoline engine exhaust. Int J Cancer 111(2):286–292. 10.1002/ijc.20263 15197784 10.1002/ijc.20263

[9] Lynge E, Andersen A, Nilsson R et al (1997) Risk of cancer and exposure to gasoline vapors. Am J Epidemiol 145(5):449–458. 10.1093/oxfordjournals.aje.a009127 9048519 10.1093/oxfordjournals.aje.a009127

[10] Lagorio S, Forastiere F, Iavarone I et al (1994) Mortality of filling station attendants. Scand J Work Environ Health 20(5):331–338. 10.5271/sjweh.1389 7863296 10.5271/sjweh.1389

[11] Soll-Johanning H, Bach E, Olsen JH, Tüchsen F (1998) Cancer incidence in urban bus drivers and tramway employees: a retrospective cohort study. Occup Environ Med 55(9):594–598. 10.1136/oem.55.9.594 9861180 10.1136/oem.55.9.594 PMC1757639

[12] Soll-Johanning H, Bach E (2004) Occupational exposure to air pollution and cancer risk among Danish urban mail carriers. Int Arch Occup Environ Health 77(5):351–356. 10.1007/s00420-004-0510-9 15108001 10.1007/s00420-004-0510-9

[13] Ancona C, Badaloni C, Mataloni F et al (2015) Mortality and morbidity in a population exposed to multiple sources of air pollution: a retrospective cohort study using air dispersion models. Environ Res 137:467–474. 10.1016/j.envres.2014.10.036 25701728 10.1016/j.envres.2014.10.036

[14] Raaschou-Nielsen O, Pedersen M, Stafoggia M et al (2017) Outdoor air pollution and risk for kidney parenchyma cancer in 14 European cohorts. Int J Cancer 140(7):1528–1537. 10.1002/ijc.30587 28006861 10.1002/ijc.30587

[15] Turner MC, Krewski D, Diver WR et al (2017) Ambient air pollution and cancer mortality in the cancer prevention study II. Environ Health Perspect 125(8):087013. 10.1289/EHP1249 28886601 10.1289/EHP1249 PMC5783657

[16] Gandini M, Scarinzi C, Bande S et al (2018) Long term effect of air pollution on incident hospital admissions: results from the Italian Longitudinal Study within LIFE MED HISS project. Environ Int 121(Pt 2):1087–1097. 10.1016/j.envint.2018.10.020 30366659 10.1016/j.envint.2018.10.020

[17] Coleman NC, Burnett RT, Ezzati M, Marshall JD, Robinson AL, Pope CA (2020) Fine particulate matter exposure and cancer incidence: analysis of SEER cancer registry data from 1992–2016. Environ Health Perspect 128(10):107004. 10.1289/EHP7246 33035119 10.1289/EHP7246 PMC7546438

[18] Raaschou-Nielsen O, Andersen ZJ, Hvidberg M et al (2011) Air pollution from traffic and cancer incidence: a Danish cohort study. Environ Health 10:67. 10.1186/1476-069X-10-67 21771295 10.1186/1476-069X-10-67 PMC3157417

[19] Cohen G, Levy I, Yuval et al (2017) Long-term exposure to traffic-related air pollution and cancer among survivors of myocardial infarction: a 20-year follow-up study. Eur J Prev Cardiol 24(1):92–102. 10.1177/2047487316669415 27625155 10.1177/2047487316669415

[20] Cohen G, Levy I, Yuval et al (2018) Chronic exposure to traffic-related air pollution and cancer incidence among 10,000 patients undergoing percutaneous coronary interventions: a historical prospective study. Eur J Prev Cardiol 25(6):659–670. 10.1177/2047487318760892 29482439 10.1177/2047487318760892

[21] Page MJ, McKenzie JE, Bossuyt PM et al (2021) The PRISMA 2020 statement: an updated guideline for reporting systematic reviews. BMJ. 10.1136/bmj.n71 33782057 10.1136/bmj.n71 PMC8005924

[22] Hamroun A, Camier A, Bigna JJ, Glowacki F (2021) Impact of air pollution on renal outcomes: a systematic review and meta-analysis protocol. BMJ Open 11(1):e041088. 10.1136/bmjopen-2020-041088 33455930 10.1136/bmjopen-2020-041088 PMC7813312

[23] Tang L, Wang QY, Cheng ZP, Hu B, Liu JD, Hu Y (2016) Air pollution and venous thrombosis: a meta-analysis. Sci Rep 6(1):32794. 10.1038/srep32794 27600652 10.1038/srep32794 PMC5013712

[24] Mustafic H, Jabre P, Caussin C et al (2012) Main air pollutants and myocardial infarction: a systematic review and meta-analysis. JAMA 307(7):713–721. 10.1001/jama.2012.126 22337682 10.1001/jama.2012.126

[25] Shah ASV, Langrish JP, Nair H et al (2013) Global association of air pollution and heart failure: a systematic review and meta-analysis. Lancet Lond Engl 382(9897):1039–1048. 10.1016/S0140-6736(13)60898-3 10.1016/S0140-6736(13)60898-3 PMC3809511 23849322

[26] Riant M, Meirhaeghe A, Giovannelli J et al (2018) Associations between long-term exposure to air pollution, glycosylated hemoglobin, fasting blood glucose and diabetes mellitus in northern France. Environ Int 120:121–129. 10.1016/j.envint.2018.07.034 30077944 10.1016/j.envint.2018.07.034

[27] Stieb DM, Berjawi R, Emode M et al (2021) Systematic review and meta-analysis of cohort studies of long term outdoor nitrogen dioxide exposure and mortality. PLoS One 16(2):e0246451. 10.1371/journal.pone.0246451 33539450 10.1371/journal.pone.0246451 PMC7861378

[28] DerSimonian R, Kacker R (2007) Random-effects model for meta-analysis of clinical trials: an update. Contemp Clin Trials 28(2):105–114. 10.1016/j.cct.2006.04.004 16807131 10.1016/j.cct.2006.04.004

[29] Huedo-Medina TB, Sánchez-Meca J, Marín-Martínez F, Botella J (2006) Assessing heterogeneity in meta-analysis: Q statistic or I2 index? Psychol Methods 11(2):193–206. 10.1037/1082-989X.11.2.193 16784338 10.1037/1082-989X.11.2.193

[30] Egger M, Davey Smith G, Schneider M, Minder C (1997) Bias in meta-analysis detected by a simple, graphical test. BMJ 315(7109):629–634. 10.1136/bmj.315.7109.629 9310563 10.1136/bmj.315.7109.629 PMC2127453

[31] Duval S, Tweedie R (2000) Trim and fill: a simple funnel-plot-based method of testing and adjusting for publication bias in meta-analysis. Biometrics 56(2):455–463. 10.1111/j.0006-341x.2000.00455.x 10877304 10.1111/j.0006-341x.2000.00455.x

[32] Hvidtfeldt UA, Taj T, Chen J et al (2022) Long term exposure to air pollution and kidney parenchyma cancer—Effects of low-level air pollution: a Study in Europe (ELAPSE). Environ Res 215(Pt 2):114385. 10.1016/j.envres.2022.114385 36154858 10.1016/j.envres.2022.114385

[33] Michelozzi P, Fusco D, Forastiere F, Ancona C, Dell’Orco V, Perucci CA (1998) Small area study of mortality among people living near multiple sources of air pollution. Occup Environ Med 55(9):611–615. 10.1136/oem.55.9.611 9861183 10.1136/oem.55.9.611 PMC1757631

[34] García-Pérez J, Fernández-Navarro P, Castelló A et al (2013) Cancer mortality in towns in the vicinity of incinerators and installations for the recovery or disposal of hazardous waste. Environ Int 51:31–44. 10.1016/j.envint.2012.10.003 23160082 10.1016/j.envint.2012.10.003

[35] Cong X (2018) Air pollution from industrial waste gas emissions is associated with cancer incidences in Shanghai, China. Environ Sci Pollut Res Int 25(13):13067–13078. 10.1007/s11356-018-1538-9 29484620 10.1007/s11356-018-1538-9

[36] Zare Sakhvidi MJ, Lequy E, Goldberg M, Jacquemin B (1987) Air pollution exposure and bladder, kidney and urinary tract cancer risk: a systematic review. Environ Pollut Barking Essex 2020(267):115328. 10.1016/j.envpol.2020.115328 10.1016/j.envpol.2020.115328 32871482

[37] Cani M, Turco F, Butticè S et al (2023) How Does environmental and occupational exposure contribute to carcinogenesis in genitourinary and lung cancers? Cancers 15(10):2836. 10.3390/cancers15102836 37345174 10.3390/cancers15102836 PMC10216822

[38] Loomis D, Grosse Y, Lauby-Secretan B et al (2013) The carcinogenicity of outdoor air pollution. Lancet Oncol 14(13):1262–1263. 10.1016/s1470-2045(13)70487-x 25035875 10.1016/s1470-2045(13)70487-x

[39] Youogo LMAK, Parent ME, Hystad P, Villeneuve PJ (2022) Ambient air pollution and prostate cancer risk in a population-based Canadian case-control study. Environ Epidemiol 6(4):e219. 10.1097/EE9.0000000000000219 35975163 10.1097/EE9.0000000000000219 PMC9374191

[40] Péry ARR, Brochot C, Hoet PHM, Nemmar A, Bois FY (2009) Development of a physiologically based kinetic model for 99m-technetium-labelled carbon nanoparticles inhaled by humans. Inhal Toxicol 21(13):1099–1107. 10.3109/08958370902748542 19814607 10.3109/08958370902748542

[41] Elder A, Oberdörster G (2006) Translocation and effects of ultrafine particles outside of the lung. Clin Occup Environ Med 5(4):785–796. 10.1016/j.coem.2006.07.003 17110292 10.1016/j.coem.2006.07.003

[42] Elder A, Gelein R, Silva V et al (2006) Translocation of inhaled ultrafine manganese oxide particles to the central nervous system. Environ Health Perspect 114(8):1172–1178. 10.1289/ehp.9030 16882521 10.1289/ehp.9030 PMC1552007

[43] Kreyling WG, Semmler-Behnke M, Seitz J et al (2009) Size dependence of the translocation of inhaled iridium and carbon nanoparticle aggregates from the lung of rats to the blood and secondary target organs. Inhal Toxicol 21(Suppl 1):55–60. 10.1080/08958370902942517 19558234 10.1080/08958370902942517

[44] Waly MI, Ali BH, Nemmar A (2013) Acute effects of diesel exhaust particles and cisplatin on oxidative stress in cultured human kidney (HEK 293) cells, and the influence of curcumin thereon. Toxicol Vitro Int J Publ Assoc BIBRA 27(8):2299–2304. 10.1016/j.tiv.2013.09.023 10.1016/j.tiv.2013.09.023 24113306

[45] Nemmar A, Karaca T, Beegam S et al (2016) Prolonged pulmonary exposure to diesel exhaust particles exacerbates renal oxidative stress, inflammation and DNA damage in mice with adenine-induced chronic renal failure. Cell Physiol Biochem Int J Exp Cell Physiol Biochem Pharmacol 38(5):1703–1713. 10.1159/000443109 10.1159/000443109 27160713

[46] Aztatzi-Aguilar OG, Uribe-Ramírez M, Narváez-Morales J, De Vizcaya-Ruiz A, Barbier O (2016) Early kidney damage induced by subchronic exposure to PM2.5 in rats. Part Fibre Toxicol 13(1):68. 10.1186/s12989-016-0179-8 27955691 10.1186/s12989-016-0179-8 PMC5154051

[47] Karami S, Boffetta P, Brennan P et al (2011) Renal cancer risk and occupational exposure to polycyclic aromatic hydrocarbons and plastics. J Occup Environ Med Am Coll Occup Environ Med 53(2):218. 10.1097/JOM.0b013e31820a40a3 10.1097/JOM.0b013e31820a40a3 PMC3065187 21270648

[48] Lue SH, Wellenius GA, Wilker EH, Mostofsky E, Mittleman MA (2013) Residential proximity to major roadways and renal function. J Epidemiol Community Health 67(8):629–634. 10.1136/jech-2012-202307 23669275 10.1136/jech-2012-202307 PMC4167787

[49] Mehta AJ, Zanobetti A, Bind MAC et al (2016) Long-term exposure to ambient fine particulate matter and renal function in older men: the veterans administration normative aging study. Environ Health Perspect 124(9):1353–1360. 10.1289/ehp.1510269 26955062 10.1289/ehp.1510269 PMC5010417

[50] Bowe B, Xie Y, Li T, Yan Y, Xian H, Al-Aly Z (2018) Particulate matter air pollution and the risk of incident CKD and progression to ESRD. J Am Soc Nephrol JASN 29(1):218–230. 10.1681/ASN.2017030253 28935655 10.1681/ASN.2017030253 PMC5748906

[51] Al-Aly Z, Bowe B (2020) Air pollution and kidney disease. Clin J Am Soc Nephrol CJASN 15(3):301–303. 10.2215/CJN.16031219 32125277 10.2215/CJN.16031219 PMC7057298

[52] Rausch S, Hennenlotter J, Todenhöfer T et al (2014) Impaired estimated glomerular filtration rate is a significant predictor for non-muscle-invasive bladder cancer recurrence and progression–introducing a novel prognostic model for bladder cancer recurrence. Urol Oncol 32(8):1178–1183. 10.1016/j.urolonc.2014.05.009 24962661 10.1016/j.urolonc.2014.05.009

[53] Wong G, Hayen A, Chapman JR et al (2009) Association of CKD and cancer risk in older people. J Am Soc Nephrol JASN 20(6):1341–1350. 10.1681/ASN.2008090998 19406977 10.1681/ASN.2008090998 PMC2689896

