# Supplementary figures and images for: Air pollution and kidney cancer risk: a systematic review and meta-analysis
Source: J Nephrol. 2024 Jun 24;37(7):1779–90. doi: 10.1007/s40620-024-01984-x (PMC11519201; doi:10.1007/s40620-024-01984-x)

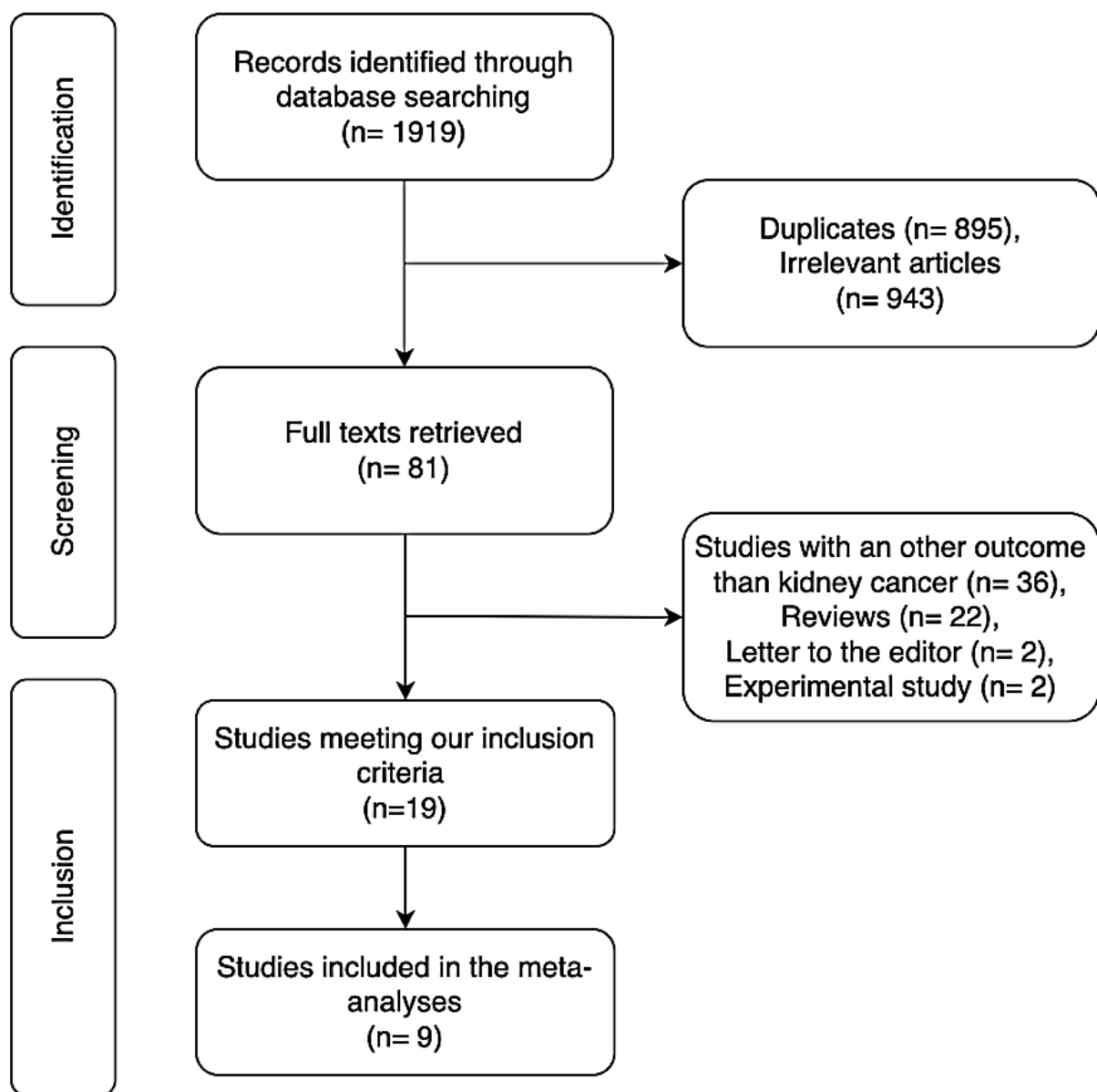

**Figure 1.** Study selection process

Supplement: Supplementary file 1 — Supplementary file1 (PDF 120 KB) [file 40620_2024_1984_MOESM1_ESM.pdf]

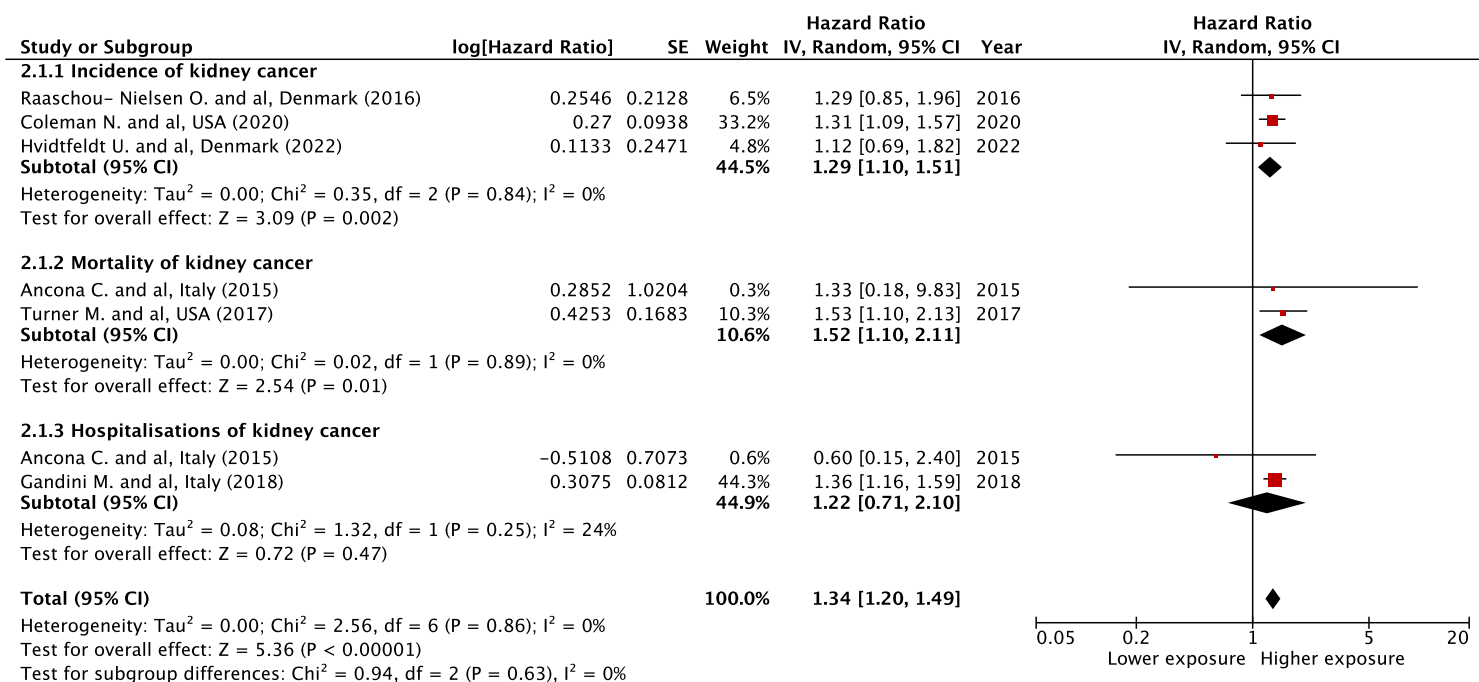

Supplement: Supplementary file 2 — Supplementary Fig. 2. Association between PM10 exposure and overall kidney cancer risk (all outcomes combined). CI, confidence interval; df, degrees of freedom; SE, standard error (PDF 530 KB) [file 40620_2024_1984_MOESM2_ESM.pdf]

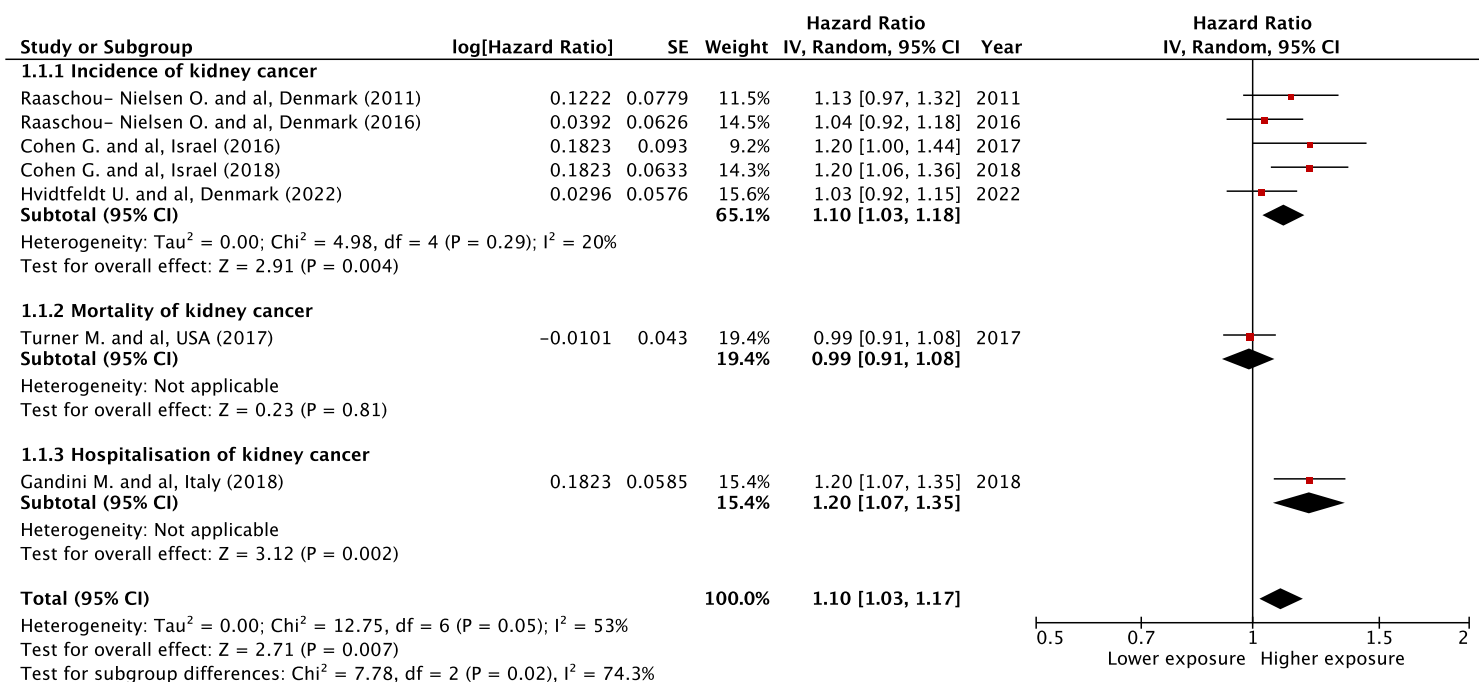

Supplement: Supplementary file 3 — Supplementary Fig. 3. Association between NO2 exposure and overall kidney cancer risk (all outcomes combined). CI, confidence interval; df, degrees of freedom; SE, standard error (PDF 515 KB) [file 40620_2024_1984_MOESM3_ESM.pdf]

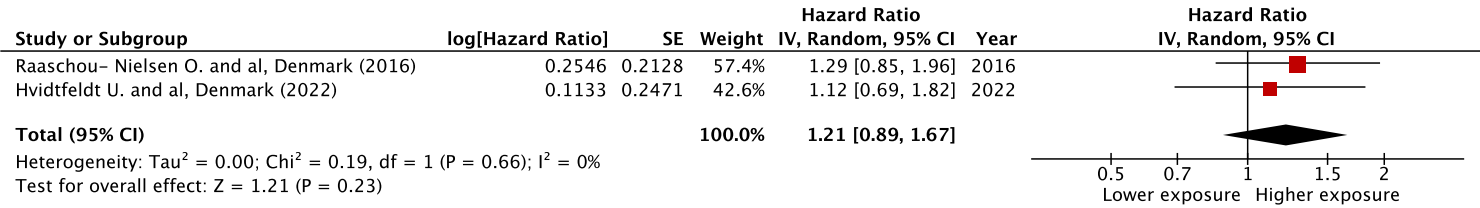

Supplement: Supplementary file 4 — Supplementary Fig. 4. Sensitivity analysis: association between PM10 exposure and the risk of kidney cancer incidence, excluding high risk of bias studies. CI, confidence interval; df, degrees of freedom; SE, standard error (PDF 179 KB) [file 40620_2024_1984_MOESM4_ESM.pdf]

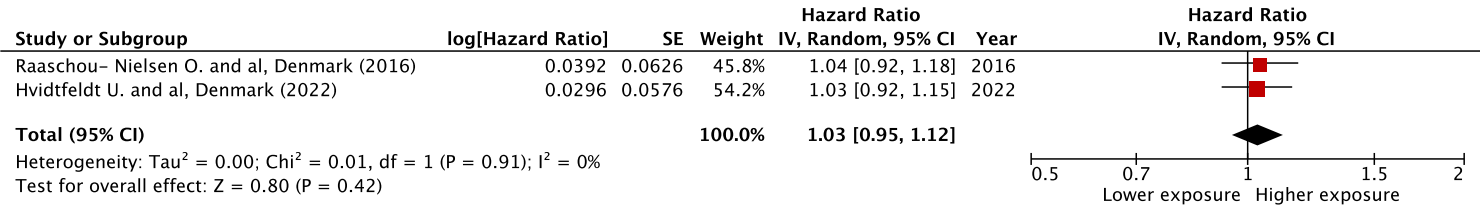

Supplement: Supplementary file 5 — Supplementary Fig. 5. Sensitivity analysis: association between NO2 exposure and the risk of kidney cancer incidence, excluding high risk of bias studies. CI, confidence interval; df, degrees of freedom; SE, standard error (PDF 180 KB) [file 40620_2024_1984_MOESM5_ESM.pdf]

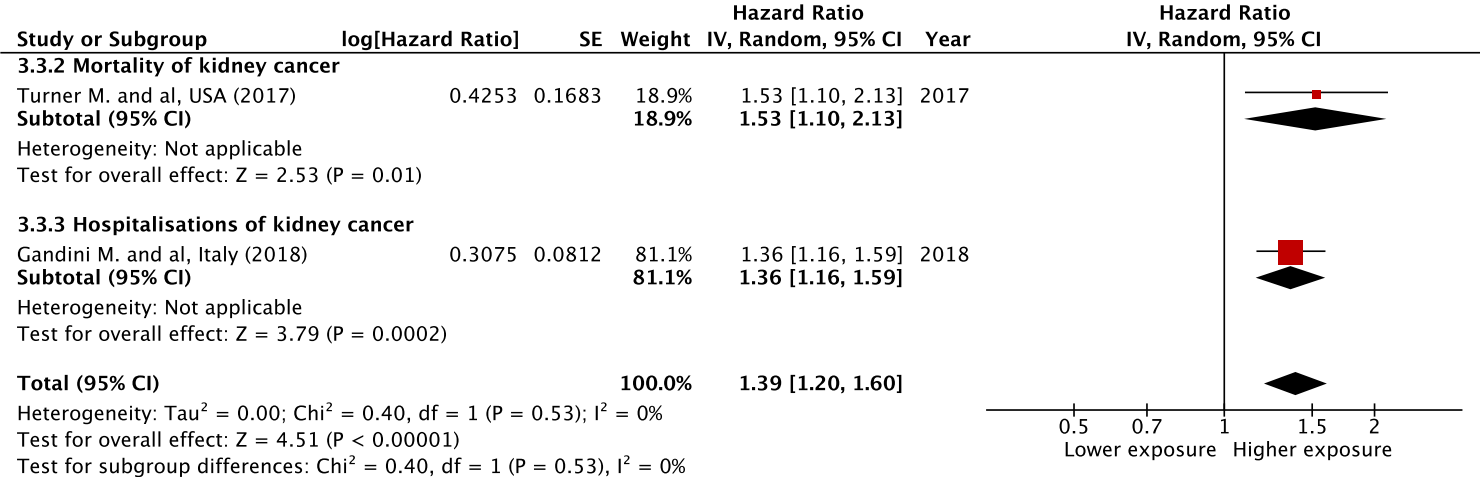

Supplement: Supplementary file 6 — Supplementary Fig. 6. Sensitivity analysis: association between PM10 exposure and the risk of kidney cancer-related morbidity/mortality (hospitalization or death), excluding high risk of bias studies. CI, confidence interval; df, degrees of freedom; SE, standard error (PDF 304 KB) [file 40620_2024_1984_MOESM6_ESM.pdf]

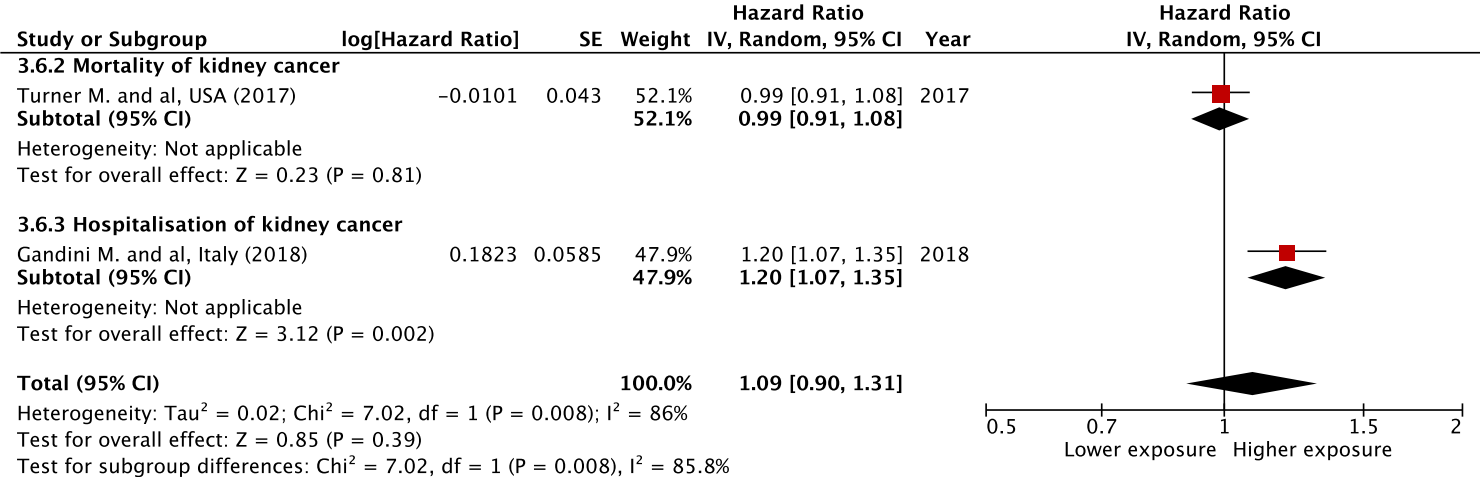

Supplement: Supplementary file 7 — Supplementary Fig. 7. Sensitivity analysis: association between NO2 exposure and the risk of kidney cancer-related morbidity/mortality (hospitalization or death), excluding high risk of bias studies. CI, confidence interval; df, degrees of freedom; SE, standard error (PDF 310 KB) [file 40620_2024_1984_MOESM7_ESM.pdf]

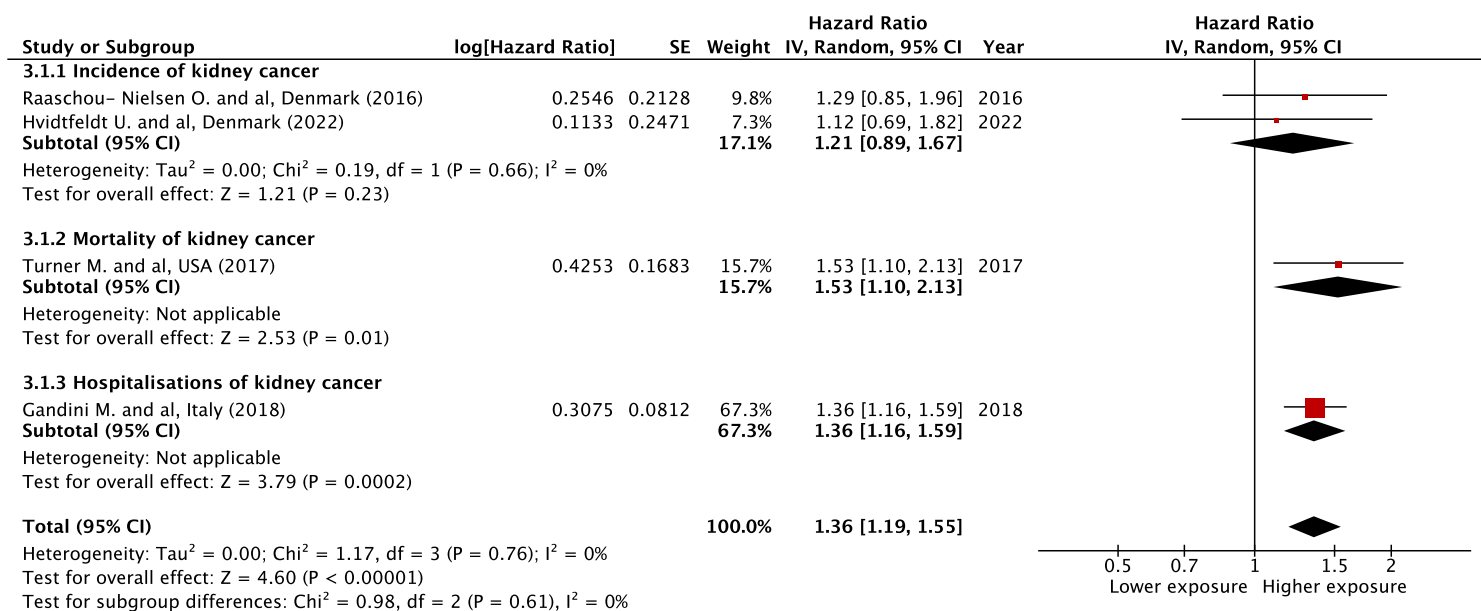

Supplement: Supplementary file 8 — Supplementary Fig. 8. Sensitivity analysis: association between PM10 exposure and overall kidney cancer risk (all outcomes combined), excluding high risk of bias studies. CI, confidence interval; df, degrees of freedom; SE, standard error (PDF 429 KB) [file 40620_2024_1984_MOESM8_ESM.pdf]

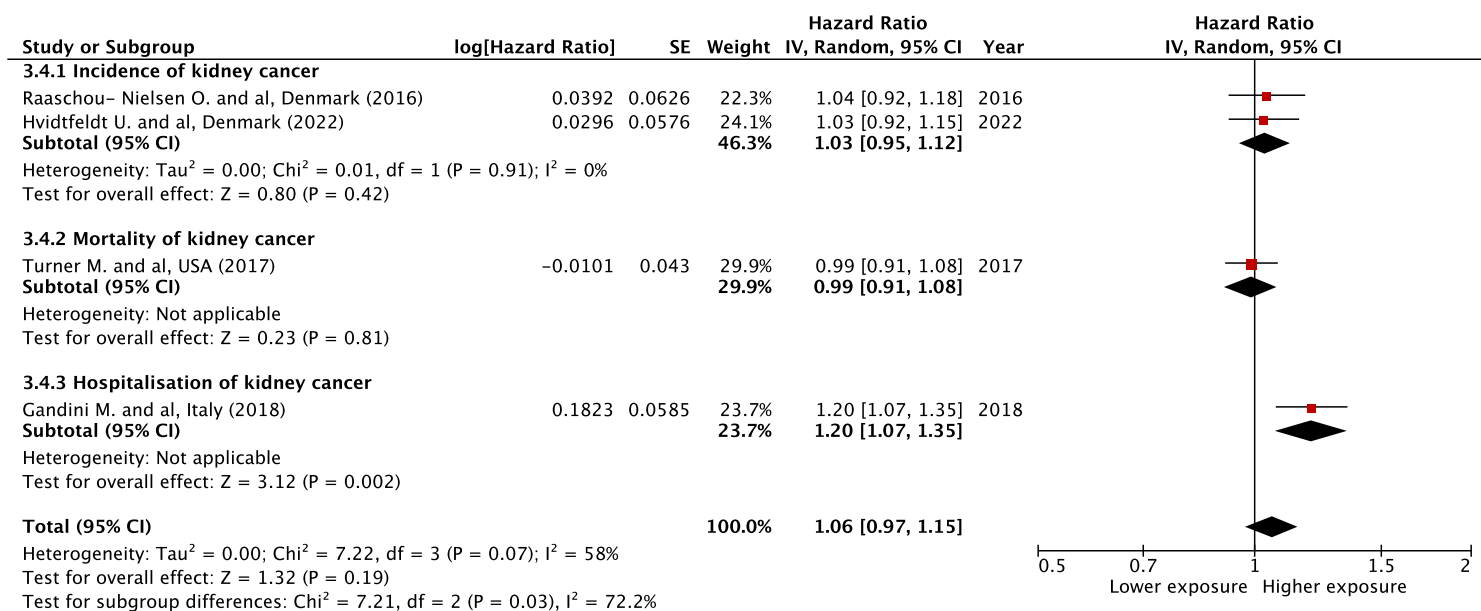

Supplement: Supplementary file 9 — Supplementary Fig. 9. Sensitivity analysis: association between NO2 exposure and overall kidney cancer risk (all outcomes combined), excluding high risk of bias studies. CI, confidence interval; df, degrees of freedom; SE, standard error (PDF 433 KB) [file 40620_2024_1984_MOESM9_ESM.pdf]

**A**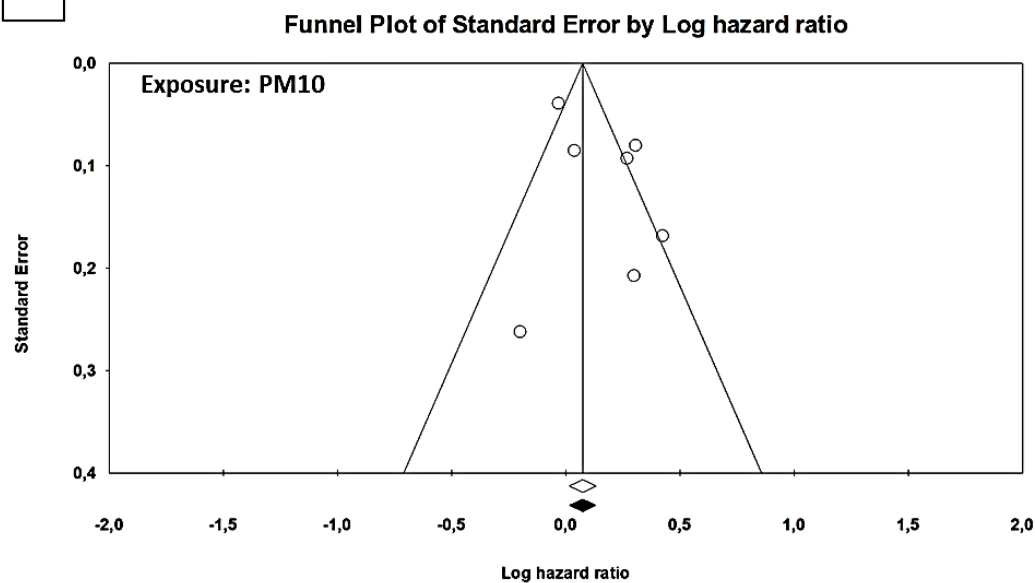

Supplement: Supplementary file 10 — Supplementary Fig. 10. Funnel plots (Trim and fill analysis) regarding PM10 (A) and NO2 (B) analyses. Unfilled and black filled circles correspond to the observed and imputed studies. Unfilled and black filled diamond shapes correspond to the observed and imputed point estimates (log hazard ratio). (PDF 136 KB) [file 40620_2024_1984_MOESM10_ESM.pdf]
