# Supplementary material for: Air pollution and kidney cancer risk: a systematic review and meta-analysis
Source: J Nephrol. 2024 Jun 24;37(7):1779–90. doi: 10.1007/s40620-024-01984-x (PMC11519201; doi:10.1007/s40620-024-01984-x)
Supplement: Supplementary file 11 — Supplementary file11 (PDF 71 KB) [file 40620_2024_1984_MOESM11_ESM.pdf]

Supplementary Table 1. Generic search strategy on EMBASE, PubMed, Web of science, Cochrane Library and CINAHL database from inception to 23 March 2023

Search	Search terms
#1	« Air pollution » OR « Air pollutant » OR « Pollution » OR « air » OR « atmosphere » OR « atmospheric » OR « atmospheric pollution » OR « particulate matter » OR « ozone » OR « carbon monoxide » OR « sulfur dioxide » OR « nitrogen dioxide » OR « PM2.5 » OR « PMcoarse» OR « PM10 » OR « NO » OR « NOx » OR « SO2 » OR « CO »
#2	« Kidney » OR « Renal» OR « chronic kidney disease » OR « renal dysfunction » OR « kidney disease » OR « end-stage renal disease » OR « kidney failure » OR « renal failure » OR « renal dialysis » OR « hemodialysis » OR « proteinuria » OR « albuminuria » OR « nephrotic syndrome » OR « nephrotic » OR « membranous nephropathy » OR « idiopathic membranous nephropathy » OR « kidney graft failure » OR « kidney transplant failure » OR « kidney cancer » OR « kidney neoplasm » OR « malignancy » OR « malignant neoplasm of kidney» OR « Glomerular filtration rate » OR « GFR » OR « eGFR » OR « MDRD » OR « CKD-EPI »
#3	#1 AND #2

Supplementary Table 2. Methodological quality assessment criteria (modified Newcastle-Ottawa scale[24])

Type of bias	Level of bias	Assessment criteria
Exposure assessment bias	High	Use of exposure as a surrogate for air pollution (e.g. distance to road, personal recall)<br>Use of single fixed monitoring system<br>Use of 2 or less fixed monitoring systems
	Medium	Use of 3 or more fixed monitoring systems<br>Use of atmospheric dispersion models (land use regression models)<br>Use of satellite/remote sensing
	Low	Personal exposure measurement<br>Combination of 2 or more of the above (providing at least one moderate level of bias)
Detection bias	High	Health outcomes not based on International Classification of Diseases diagnosis codes nor on clinically confirmed outcomes
Selection bias	High	Unrepresentative study populations
Adjustment for confounders	High	Studies with no adjustment for at least three of the following main confounders—long-term trends, seasonality, weather, population characteristics, and lifestyle factors (such as smoking status and alcohol consumption)
Global risk of bias	High	At least two components at high risk of bias
	Medium	Only one component at high risk of bias
	Low	All four components at low risk of bias
